# The gravity dual of Rényi entropy

**DOI:** 10.1038/ncomms12472

**Published:** 2016-08-12

**Authors:** Xi Dong

**Affiliations:** 1 School of Natural Sciences, Institute for Advanced Study, Princeton, New Jersey 08540, USA

## Abstract

A remarkable yet mysterious property of black holes is that their entropy is proportional to the horizon area. This area law inspired the holographic principle, which was later realized concretely in gauge-gravity duality. In this context, entanglement entropy is given by the area of a minimal surface in a dual spacetime. However, discussions of area laws have been constrained to entanglement entropy, whereas a full understanding of a quantum state requires Rényi entropies. Here we show that all Rényi entropies satisfy a similar area law in holographic theories and are given by the areas of dual cosmic branes. This geometric prescription is a one-parameter generalization of the minimal surface prescription for entanglement entropy. Applying this we provide the first holographic calculation of mutual Rényi information between two disks of arbitrary dimension. Our results provide a framework for efficiently studying Rényi entropies and understanding entanglement structures in strongly coupled systems and quantum gravity.

## Abstract (web summary)

The discovery that the entropy of black holes is given by their horizon area inspired the holographic principle and led to gauge-gravity duality. Here, the author shows that all Rényi entropies satisfy a similar area law in holographic theories and are given by the areas of dual cosmic branes.

## Graphical abstract

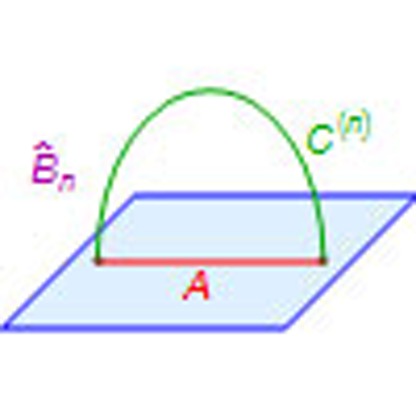

One of the most remarkable discoveries in fundamental physics is that black holes carry entropy with an amount equal to a quarter of the horizon area in Planck units^1^,^2^,^3^:


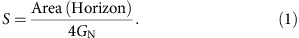


Here *G*_N_ denotes Newton's constant. The property that gravitational entropies satisfy an area law, however, is not restricted to black holes. It was elegantly generalized by Ryu and Takayanagi^4^ in the context of gauge/gravity duality, an exact equivalence between certain strongly coupled quantum field theories (QFTs) and weakly coupled gravitational theories in one higher dimensions^5^,^6^,^7^. In this context, they proposed that the von Neumann entropy, also known as the entanglement entropy, of any spatial region *A* at a moment of time-reflection symmetry in the boundary QFT is determined by the area of a codimension-2 minimal surface in the dual spacetime:


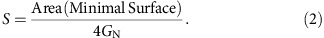


The minimal surface is constrained to be at a moment of time-reflection symmetry in the bulk and homologous to the entangling region *A*. In particular, this means that the minimal surface is anchored at the entangling surface ∂*A*. Here we follow the standard terminology of referring to the dual spacetime in which the gravitational theory lives as the bulk, and identify the spacetime in which the QFT lives with the asymptotic boundary of the bulk spacetime.

This elegant prescription for holographic entanglement entropy was initially proven in the special case of spherical entangling regions in the vacuum state of a conformal field theory (CFT), by employing a *U*(1) symmetry to map the problem to one of finding the thermal entropy of the CFT on a hyperboloid^8^. The latter problem was then solved by gauge/gravity duality, which tells us that the thermal state of the CFT is dual to a hyperbolic black hole in the bulk, and the thermal entropy is given by the area of the black hole horizon according to equation (1). Not surprisingly, this horizon is mapped back to the Ryu–Takayanagi minimal surface in the original problem.

In more general cases, there is no *U*(1) symmetry to facilitate such a derivation. Nonetheless, Lewkowycz and Maldacena^9^ overcame this difficulty and showed that the Ryu–Takayanagi prescription (2) follows from gauge/gravity duality, by applying the replica trick and generalizing the Euclidean method developed in refs 2, 10 of calculating gravitational entropies to cases without a *U*(1) symmetry. Similar techniques were used in refs 11, 12, 13 to generalize the Ryu–Takayanagi prescription to cases where the bulk theory involves higher derivative gravity, and used in ref. 14 to find quantum corrections to the prescription.

Discussions of area laws such as equations (1) and (2) have so far been constrained to the von Neumann entropy. The main goal of this paper is to generalize these laws to Rényi entropies^15^, which are labelled by an index *n* and defined in terms of the density matrix *ρ* of the entangling region as


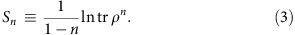


In the *n*→1 limit, we recover the von Neumann entropy *S*≡−Tr(*ρ* ln *ρ*).

Although Rényi entropies are often introduced as a one-parameter generalization of the von Neumann entropy, they are much easier to experimentally measure and numerically study (see, for example, ref. 16 for recent progress in measurements). They also contain richer physical information about the entanglement structure of a quantum state. In particular, the knowledge of Rényi entropies for all *n* allows one to determine the whole entanglement spectrum (the set of eigenvalues of *ρ*). Rényi entropies have been extensively studied by numerical methods^17^, in spin chains^18^, in tensor networks^19^, in free field theories^20^, in two-dimensional CFTs^21^,^22^,^23^,^24^,^25^,^26^,^27^,^28^ or higher^29^,^30^,^31^,^32^,^33^,^34^,^35^,^36^,^37^, and in the context of gauge/gravity duality^38^,^39^,^40^,^41^,^42^,^43^,^44^,^45^. They have also been generalized to charged^46^ and supersymmetric cases^47^,^48^,^49^.

In this paper, we show that all Rényi entropies satisfy a similar area law in holographic theories. As we will see, the gravity dual of Rényi entropy is a cosmic brane. This provides a simple geometric prescription for holographic Rényi entropies, generalizing the Ryu–Takayanagi prescription (2) for entanglement entropy. It is important to distinguish our area law from a universal feature of entanglement and Rényi entropies, which is that the most ultraviolet divergent part of these entropies is proportional to the area of the entangling surface in any QFT^50^,^51^ (see also ref. 52 and references therein for area-law bounds on entanglement entropy). Our area law reproduces and goes beyond this universal feature in that it is an exact prescription that applies to both finite and divergent parts of Rényi entropies, and it serves as a useful criterion in distinguishing theories with a gravitational dual from those without.

## Results

### An area-law prescription for Rényi entropy

Our main result is that a derivative of holographic Rényi entropy *S*_*n*_ with respect to the Rényi index *n* satisfies an area law. It is given by a quarter of the area in Planck units of a bulk codimension-2 cosmic brane homologous to the entangling region:


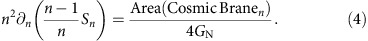


Here the subscript *n* on the cosmic brane denotes that its brane tension as a function of *n* is given by


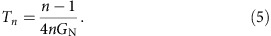


**Figure 1:**
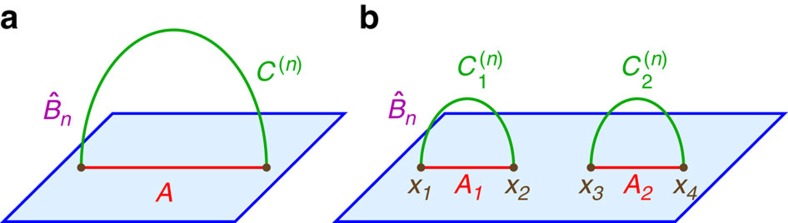
Two examples of cosmic branes as the gravity dual of Rényi entropy. The entangling region *A* (red) is either (**a**) connected or (**b**) disconnected. In each case a strongly coupled QFT on the plane (blue) has a holographic dual description in terms of a gravitational theory in the bulk spacetime above the plane. The cosmic brane *C*^(*n*)^ (green) is anchored at the entangling surface ∂*A* (brown) and backreacts on the bulk geometry 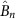, although the backreaction is difficult to show in the figure. The Rényi entropy is determined by the area of the cosmic brane. As the Rényi index *n* approaches 1 the cosmic brane become a non-backreacting minimal surface, reproducing the Ryu–Takayanagi prescription for entanglement entropy.

As shown in Fig. 1, the cosmic brane is analogous to the Ryu–Takayanagi minimal surface, except that it backreacts on the ambient geometry by creating a conical deficit angle^53^


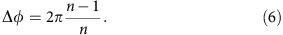


A useful way of obtaining the bulk geometry with a cosmic brane is to find the classical solution to the equations of motion resulting from the total (Euclidean) action 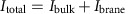, where the bulk action is the Einstein–Hilbert action with matter


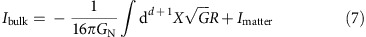


and the brane action is the Nambu–Goto action 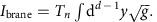 Here *X*^*μ*^, *G*_*μv*_ and *R* denote the coordinates, metric and Ricci scalar in the bulk, *y*^*i*^ and *g*_*ij*_ denote the coordinates and induced metric on the brane, and *d*+1 is the bulk spacetime dimension. The action *I*_total_ governs both the bulk metric *G*_*μv*_ and the embedding map *y*^*i*^(*X*) of the brane. Finding these generally involves solving partial differential equations. In cases where *I*_total_ admits more than one classical solutions, we choose the dominant solution which has the smallest bulk action *I*_bulk_ (not including *I*_brane_ for reasons that will become clear when we prove our area law).

In the *n*→1 limit, the cosmic brane becomes tensionless and reduces to a probe brane. It no longer backreacts on the geometry and instead settles at the location of the minimal surface. It is manifest that our area law (4) reduces to the Ryu–Takayanagi prescription (2) for entanglement entropy in the *n*→1 limit. Therefore, our result is a one-parameter generalization of the Ryu–Takayanagi prescription.

Our area law (4) suggests that a close variant of the Rényi entropy


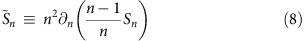


is a more natural candidate for generalizing the von Neumann entropy in that 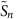 precisely satisfies an area law. In terms of the density matrix *ρ*, it is defined by


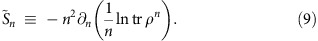


It is illuminating to rewrite this as a standard thermodynamic relation


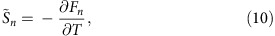


with the free energy 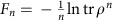 and temperature *T*=1/*n*. This can be made precise by noting that tr*ρ*^*n*^ is the canonical partition function with respect to the modular Hamiltonian *K* ≡−ln *ρ*. Similar relations between the Rényi entropy and free energy have been discussed in refs 54, 55.

In this language, it is useful to compare and contrast our area law (4) with the prescription for holographic Rényi entropies in ref. 9. There a method was developed for calculating the free energy *F*_*n*_ in terms of an on-shell action in the bulk, but our result here goes further in showing that the natural entropy 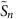 defined by equation (10) localizes on a codimension-2 surface (the cosmic brane) in the bulk and is determined by the surface area in Planck units. This localization effect is both conceptually interesting and practically useful. It allows us to calculate Rényi entropies in nontrivial situations as we will see later in an example. It also agrees with the recent progress in understanding modular Hamiltonians holographically^56^.

The natural entropy 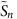 is known to be non-negative for *n*>0 in all quantum systems^54^. This fact is made manifest by our area law (4). From the quantum information perspective, it is worth studying 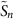 as a new measure of quantum entanglement.

It is interesting to note that an integrated version of equation (4) was used in ref. 39 to calculate Rényi entropies for the special case of spherical entangling regions in a CFT.

It is straightforward to generalize our result (4) to theories dual to higher derivative gravity along the directions of refs 11, 12, 13. We replace the right-hand side of equation (4) by the Wald entropy^57^,^58^,^59^ evaluated on a cosmic brane that produces—in the higher derivative gravity—a conical deficit angle given by equation (6) for *n*≠1, and the extrinsic curvature corrections derived in refs 11, 12, 13 appear in the *n*→1 limit. Similarly, using the methods of refs 14, 60 we may include quantum corrections to our result in terms of the bulk Rényi entropy across the cosmic brane.

### Derivation via the holographic replica trick

We derive the area-law prescription (4) for holographic Rényi entropies by applying the replica trick in the context of gauge/gravity duality. We follow the method used in ref. 9 for deriving the Ryu–Takayanagi prescription.

Let us start by recalling that the Rényi entropy (3) of integer index *n*>1 is simply determined by the partition function of the QFT on a branched cover *M*_*n*_, defined by taking *n* copies of the original Euclidean spacetime *M*_1_ on which the QFT lives with a cut along the entangling region and gluing them along the cuts in a cyclic order. This may be written as


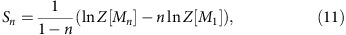


where *Z*[*M*_*n*_] and *Z*[*M*_1_] denote the partition function on the branched cover and original spacetime, respectively. For holographic QFTs, we may calculate *Z*[*M*_*n*_] by finding the dominant bulk solution *B*_*n*_ whose asymptotic boundary is *M*_*n*_. In the so-called large *N* limit where the bulk physics is classical, we have


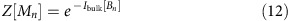


where *I*_bulk_[*B*_*n*_] denotes the on-shell action of the bulk solution. Here we work in the Euclidean signature as in ref. 9 by assuming that the entangling region is at a moment of time-reflection symmetry, and leave covariant generalizations along the direction of ref. 61 to future work.

The branched cover *M*_*n*_ has a manifest 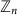 symmetry that cyclically permutes the *n* replicas. As in ref. 9, we assume that this 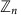 replica symmetry is not spontaneously broken in the dominant bulk solution *B*_*n*_. There is no known example of replica symmetry breaking in the context of gauge/gravity duality although it remains an interesting possibility for further study. Taking a quotient by the 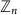 replica symmetry, we define an orbifold


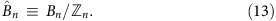


Since the bulk action is local, we may write


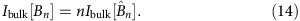


Substituting equations (12) and (14) into equation (11), we find


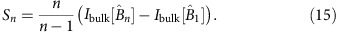


Generally, the branched cover *M*_*n*_ and its bulk dual *B*_*n*_ are meaningful only for integer *n*. Nonetheless, it is possible to analytically continue the orbifold 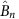 to non-integer *n*. The prescription is to use the cosmic brane introduced earlier and find the classical solution resulting from the action *I*_total_. To see that this gives the same solution as the orbifold (13) for integer *n*, we note that they have the same codimension-2 conical singularity with the deficit angle (6). For the orbifold this results from the regularity of the parent space which is required to satisfy the equations of motion everywhere in the bulk, and the codimension-2 singularity of the orbifold consists of the fixed points of the 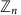 replica symmetry (see ref. 62 for a detailed discussion of the homology constraint).

Having analytically continued the orbifold 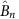 to non-integer *n*, we may now use equation (15) to calculate the Rényi entropy. It is worth emphasizing that the on-shell action 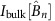 appearing in equation (15) does not include any contribution from the cosmic brane. This is manifest for integer *n* because there is no significant contribution from the 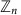 fixed points to the left-hand side of equation (14), and the same is true for its right-hand side. In this sense the cosmic brane is a useful auxiliary tool for generating the relevant conical solutions.

To derive our area law (4) for holographic Rényi entropies, we note that equation (15) implies


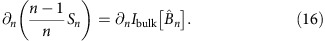


To evaluate this, we view 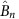 as a family of classical solutions to the equations of motion with varying boundary conditions at the cosmic brane. The variation of the on-shell action is therefore a boundary term. For Einstein gravity (7), we find explicitly


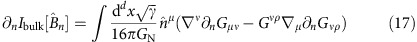


where we evaluate the integral on a thin codimension-1 tube around the cosmic brane, taking the thickness to zero at the end of the calculation. Here *x*^*α*^ and γ_*αβ*_ denote the coordinates and induced metric on this tube, and 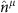 is the unit normal vector pointing away from the cosmic brane. The boundary term (17) was calculated in the *n*→1 limit in ref. 9 but here we keep *n* arbitrary. We may evaluate equation (17) in any coordinate system; one particular choice is to use polar coordinates *r*, *φ* to describe the 2-dimensional plane orthogonal to the cosmic brane, such that the metric near the brane is


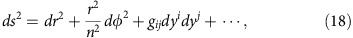


where the range of *φ* is fixed as 2*π* and *y*^*i*^ denotes the coordinates on the brane. Evaluating equation (17) in this way and using equation (16), we find the desired area law (4).

### Applications

Our area law (4) gives a simple prescription for calculating holographic Rényi entropy of any index *n* for an arbitrary entangling region. Applying this prescription is no more difficult than solving partial differential equations, which can be achieved numerically and in some cases analytically. In principle this allows one to reconstruct the whole entanglement spectrum. To demonstrate the application of our prescription in a simple example, we provide the first result for the mutual Rényi information between two spherical disks in a holographic CFT in *d* spacetime dimensions. This is illustrated in Fig. 1b for the case of *d=*2.

The Rényi entropy for a region in a QFT is ultraviolet divergent due to the entanglement between short-distance modes across the entangling surface, but the mutual Rényi information between two disjoint regions *A*_1_ and *A*_2_


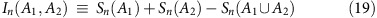


is finite and regulator-independent. In our example, *A*_1_ and *A*_2_ represent the two disks. The mutual Rényi information depends on the radii *R*_1_, *R*_2_ of the disks and the distance *D* between their centres only through the conformally invariant cross-ratio


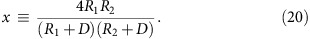


Our area law (4) reduces the problem to one of finding the bulk solution with a cosmic brane homologous to the union of two disks. There are two possible topologies for the cosmic brane: either a union of two cosmic branes 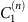, 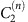 anchored at ∂*A*_1_, ∂*A*_2_ respectively as shown in Fig. 1b, or a tube connecting ∂*A*_1_ with ∂*A*_2_. We will focus on the first topology which gives the dominant bulk solution for *x*<*x*_*c*_, with *x*_*c*_ a critical cross-ratio at which the two topologies give equal *I*_bulk_ and a phase transition happens for the Rényi entropy.

In the Methods section we derive the mutual Rényi information between the two disks to linear order in *δn*≡*n*−1:


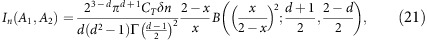


where *B* is the incomplete beta function and *C*_*T*_ is a central charge appearing in the vacuum two-point function of the stress tensor of the CFT in flat space^63^:


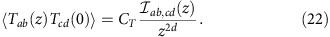


Here we have defined 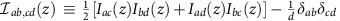 and 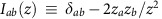. Our result (21) is derived for CFTs dual to Einstein gravity, but similar techniques can be used for higher derivative gravity (or to higher orders in *δn*).

In the special case of *d*=2, we may use *C_T_*=*c*/2π^2^ to rewrite equation (21) in terms of the central charge *c* of the Virasoro algebra and find at linear order in *δn*


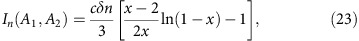


which agrees with the result of a CFT derivation in ref. 27 and short interval expansions computed in refs 38, 44, 45.

## Discussion

The area law for black holes (1) led to much progress in understanding quantum gravity. It inspired the holographic principle^64^,^65^, which states that the fundamental degrees of freedom describing any region in quantum gravity are actually encoded on its boundary. The minimal surface prescription of Ryu and Takayanagi (2) led to numerous results on the von Neumann entropy in strongly coupled systems. It also played an essential role in establishing a deep and mysterious connection between quantum entanglement and gravity^66^,^67^,^68^.

Rényi entropies, however, have until now been more difficult to study than the von Neumann entropy in strongly coupled theories, even though they are more experimentally accessible and contain richer information about a quantum state. Our area law (4) provides a framework for efficiently studying Rényi entropies in strongly coupled systems. Given the recent advances in experimentally measuring Rényi entropies, it is worth exploring whether they share similar features with the prediction of our area law in holographic theories. Progress in this direction would also provide opportunities for distinguishing holographic theories from non-holographic ones, and for eventually understanding the connection between quantum entanglement and gravity.

## Methods

### Mutual Rényi information between two disks

We provide the calculation of the Rényi entropy for the union of two disjoint disks to linear order in *δn*≡*n*−1, in the vacuum state of a holographic CFT in *d*-dimensional Minkowski space. Here disks are defined to be perfectly spherical regions in *d−*1 dimensions.

For a single disk, the Rényi entropy was calculated in ref. 39 by using the insight of ref. 8 to map the problem to one of calculating the thermal entropy of the CFT on a unit hyperboloid at temperature *T*=1/2*πn*. The holographic calculation then proceeds by finding the relevant hyperbolic black hole in the bulk. In our language, this means that we know explicitly the bulk solution with a cosmic brane homologous to a single disk.

The mutual Rényi information (19) is all that we need to understand the Rényi entropy for the union of two disks. We will express our results in terms of a conformally invariant cross-ratio *x*. To define *x* we view the line connecting the two disk centres as a coordinate axis, and it intersects the sphere *∂A*_1_ at *x*_1_, *x*_2_ and the sphere *∂A*_2_ at *x*_3_, *x*_4_, as shown in Fig. 1b for *d*=2. The cross-ratio *x* is defined in the standard way from these four points:


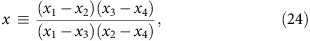


and it takes real values between 0 and 1.

We will focus on the *x*<*x*_*c*_ phase in which the dominant bulk solution contains two separate cosmic branes 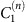, 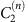 homologous to *A*_1_, *A*_2_ respectively as shown in Fig. 1b. Here *x*_*c*_ is a critical cross-ratio where a phase transition happens and the dominant bulk solution for *x*>*x*_*c*_ switches to one with a tube-like cosmic brane connecting *∂A*_1_ with *∂A*_2_. For *d*=2, the value of *x*_*c*_ is 1/2 regardless of *n*, because under the exchange of the entangling region and its complement, we exchange the two brane topologies and *x* with 1−*x*. In higher dimensions the value of *x*_*c*_ can be numerically calculated for *n*=1 as in ref. 69 but may generally depend on *n*.

In the *x*<*x*_*c*_ phase, the mutual information *I*_1_(*A*_1_, *A*_2_) vanishes according to the Ryu–Takayanagi prescription (2), but for *n*≠1 the two cosmic branes 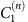, 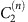 feel the backreaction of each other. Applying our area law (4) in equation (19) we find


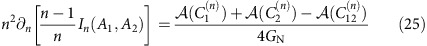



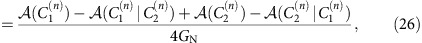


where 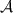 represents the area and 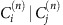 denotes the cosmic brane 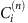 in the presence of 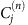. Working at linear order in *δn*, we may replace the area difference 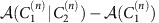 by


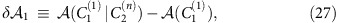


which is the area variation of the minimal surface 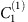 homologous to *A*_1_ due to the backreaction of 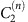. This is because the self-backreaction of the first brane is only affected by the second at higher orders in *δn*. Therefore, expanding equation (26) to linear order in *δn* we find


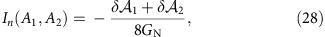


with 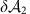 defined similarly as in equation (27).

We will calculate equation (28) in two steps. First, we find the bulk solution with a cosmic brane homologous to one of the two disks, say the first one *A*_1_. To do this we start by using a suitable conformal transformation to map *A*_1_ to the outside of the unit (*d*−2)-sphere and *A*_2_ to the inside of a (*d*−2)-sphere with radius *R*_0_, with both spheres centred at the origin. The value of *R*_0_ is determined from the cross-ratio by


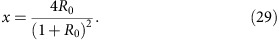


To see this, we use equation (24) with *x*_1_=1, *x*_2_=−1, *x*_3_=−*R*_0_ and *x*_4_=*R*_0_.

The next step is to conformally map the complement of *A*_1_ which is a unit disk to the (*d*−1)-dimensional unit hyperboloid 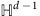. We do this by noting that the *d*-dimensional flat space metric 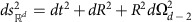 is conformally equivalent to


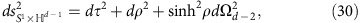


which describes 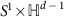 with coordinates defined by 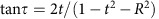 and 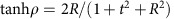. The *τ* coordinate has period 2*π*. Choosing the two disks to be on *t*=0, we find that the entangling surface of *A*_1_ is mapped to *ρ*=∞, and *A*_2_ is mapped to the region *ρ*<*ρ*_0_ with


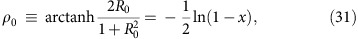


where we have used equation (29).

We may now write down the bulk geometry with a cosmic brane homologous to the first disk *A*_1_:


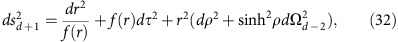


where for Einstein gravity we have


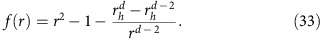


Here we work in the units where the radius of curvature in the asymptotic bulk geometry is set to 1, and *r* denotes the holographic direction in the bulk. As *r*→∞ we approach the asymptotic boundary and recover the metric (30) on 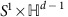 conformally. The bulk (32) is often viewed as a (Euclidean) black hole geometry with a smooth horizon at *r*=*r*_*h*_ when *τ* has period 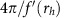, but here we fix the period of *τ* as 2*π* and attribute the conical singularity at *r*=*r*_*h*_ to a cosmic brane. Matching the conical deficit angle with equation (6) we find


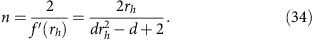


We now perform the second step of our calculation which involves finding the minimal surface homologous to the second disk *ρ*<*ρ*_0_ in the geometry (32) and calculating its area to linear order in *δn*. By spherical symmetry we may describe the minimal surface with a function *r*(*ρ*). Its area is


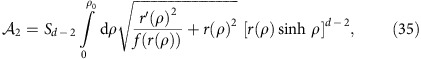


where 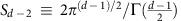 is the area of the unit (*d*−2)-sphere. The Euler–Lagrange equation from minimizing equation (35) is difficult to solve in closed form for general *n*, but for *n*=1 the minimal surface is described by


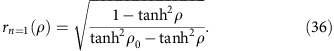


As we vary *n* away from 1, there are two potential contributions to the change of the area (35). The first is due to changing *f*(*r*) without moving the surface *r*(*ρ*), and this contribution may be directly calculated from equations (33–36):


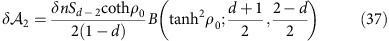


up to higher orders in *δn*. Here *B* is the incomplete beta function. The second contribution to the area change is due to moving the surface *r*(*ρ*) without changing *f*(*r*), but to linear order in *δn* this is a boundary term at *ρ*=*ρ*_0_, which may be explicitly shown to vanish.

There is a conformal 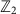 symmetry that exchanges the two disks, so they are on equal footing and 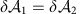. Applying equations (31) and (37) in equation (28), we find that the mutual Rényi information between the two disks is


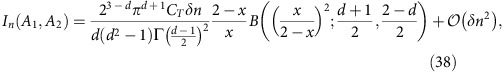


where we have used the relation^70^


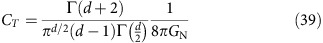


between *G*_N_ and the central charge *C*_*T*_ defined in equation (22).

### Data availability

Data sharing not applicable to this article as no datasets were generated or analysed during the current study.

## Additional information

**How to cite this article:** Dong, X. The gravity dual of Rényi entropy. *Nat. Commun.* 7:12472 doi: 10.1038/ncomms12472 (2016).
